# A Population Based Study of Seasonality of Skin and Soft Tissue Infections: Implications for the Spread of CA-MRSA

**DOI:** 10.1371/journal.pone.0060872

**Published:** 2013-04-02

**Authors:** Xiaoxia Wang, Sherry Towers, Sarada Panchanathan, Gerardo Chowell

**Affiliations:** 1 Mathematical, Computational and Modeling Sciences Center, School of Human Evolution and Social Change, Arizona State University, Tempe, Arizona, United States of America; 2 Department of Pediatrics, Maricopa Integrated Health System, Phoenix, Arizona, United States of America; 3 Department of Biomedical Informatics, Arizona State University, Tempe, Arizona, United States of America; 4 Division of Epidemiology and Population Studies, Fogarty International Center, National Institutes of Health, Bethesda, Maryland, United States of America; National Institutes of Health, United States of America

## Abstract

Methicillin resistant *Staphylococcus aureus* (MRSA) is currently a major cause of skin and soft tissue infections (SSTI) in the United States. Seasonal variation of MRSA infections in hospital settings has been widely observed. However, systematic time-series analysis of incidence data is desirable to understand the seasonality of community acquired (CA)-MRSA infections at the population level. In this paper, using data on monthly SSTI incidence in children aged 0–19 years and enrolled in Medicaid in Maricopa County, Arizona, from January 2005 to December 2008, we carried out time-series and nonlinear regression analysis to determine the periodicity, trend, and peak timing in SSTI incidence in children at different age: 0–4 years, 5–9 years, 10–14 years, and 15–19 years. We also assessed the temporal correlation between SSTI incidence and meteorological variables including average temperature and humidity. Our analysis revealed a strong annual seasonal pattern of SSTI incidence with peak occurring in early September. This pattern was consistent across age groups. Moreover, SSTIs followed a significantly increasing trend over the 4-year study period with annual incidence increasing from 3.36% to 5.55% in our pediatric population of approximately 290,000. We also found a significant correlation between the temporal variation in SSTI incidence and mean temperature and specific humidity. Our findings could have potential implications on prevention and control efforts against CA-MRSA.

## Introduction

Methicillin resistant *Staphylococcus aureus* (MRSA) is endemic in many US hospitals [1], long-term care facilities [2], and communities [3], [4]. MRSA was estimated to be associated with 125,969 hospitalizations annually in the United States from 1999 to 2000 [5]. Moreover, estimates of MRSA-related hospitalizations more than doubled from 1999 through 2005 [1]. While the rates of health care associated (HA) -MRSA infections are declining in the US [6], community acquired (CA) -MRSA infections, predominantly skin and soft tissue infections (SSTI) [7], [8], have increased markedly in the last decade in the United States [3], [9]. Because CA-MRSA infections are often acquired in the community and treated in emergency rooms and offices [8], [10], the effect of traditional transmission control measures in hospitals is limited [11]. Thus, improving our understanding of the temporal trend and seasonality patterns of CA-MRSA infections at the population level has the potential to inform surveillance and public health control strategies.

A short-term retrospective study of SSTIs diagnosed in the Pediatric Emergency Department of Johns Hopkins Hospital covering November 1, 2003 to October 31, 2005 suggested that MRSA infections could follow a seasonal pattern worth of close examination. That study reported the highest number of SSTIs during July-September [12]. Using MRSA isolates submitted to Rhode Island Hospital microbiology laboratory from January 2001 to March 2010 and the associated emergency department (ED) visits, Mermel et al. [13] reported higher CA-MRSA incidence during months July-December compared to months January-June. This pattern was more pronounced among pediatric patients. In another study, Frei et al [14] employed hospital discharge SSTIs records during 1996–2006 in the US and found peak CA-MRSA incidence in children (aged 0–19 years) occurring from May to December with variation across geographic regions in the USA. Leekha et al. [15] recently reviewed a number of epidemiological studies that have evaluated the seasonality in *S. aureus* colonization and infection and found that 31 out of 41 studies reported seasonal variation in *S. aureus*. In particular, all of the 10 studies on *S. aureus* –associated SSTIs noted its seasonal pattern with varying peak timing between summer and autumn. Also, it was pointed out that only a few of these studies have attempted to correlate seasonal patterns of *S. aureus* infections with climate-related factors such as temperature and humidity [16]–[19]. They also underscored the need to apply appropriate statistical methods (e.g., time-series analysis) to assess seasonality patterns based on incidence at the population level rather than absolute case counts. Thus, studies based on time-series analysis techniques and MRSA case counts in outpatient settings that cover a significant fraction of the population for several years are needed in order to comprehensively assess seasonality patterns and trends in MRSA infections.

In this paper, we use a population-based research data repository of SSTIs in children and youth aged 0–19 years covering the period from January 1, 2005 to December 31, 2008 to determine the temporal trend, seasonality pattern, and peak timing of MRSA infections in different age groups. We also examine the correlation between MRSA infections and potentially associated environmental factors such as average temperature and humidity.

## Materials and Methods

### Study location: Maricopa County, Arizona

Maricopa County is the third most populous local public health jurisdiction in the US, behind New York City and Los Angeles County, with a population of 3.8 million comprising 60% of Arizona's population.

### Epidemiologic Data Collection

We obtained our study data from the Center for Health Information Research (CHIR). CHIR is a university-community partnership between ASU and several Arizona providers, insurers and employers. It maintains a research data repository that integrates Arizona-based administrative, clinical and public health data across a large number of sources permitting the conduct of population-based research on residents of Arizona [20]. Specifically, we obtained data on hospitalization and outpatients visits of children and youth (age< = 19 year) who were continuously enrolled for at least 6 months in the Arizona Health Care Cost Containment System (AHCCCS) program (Medicaid) during the period January 1, 2005 to December 31, 2008. The records of all encounters with individuals aged < = 19 years and diagnosed with skin or soft tissue infection based on ICD 9 codes (680.xx–682.9x) in Maricopa County were extracted. These codes correspond to cellulitis and abscesses, but do not include superficial skin infections such as impetigo. A recurrent infection was presumed to have happened if a second visit was greater than 30 days after an initial visit while visits occurring prior to that time were presumed to be follow-up visits. These were clinical case definitions since most often cellulitis is treated without any laboratory testing and test results for abscesses are often unavailable at the time of visit. Our data comprises 51,287 patient encounters including both first-time and recurrent infections during the 4-year period, with a stationary covered population, ranging from 287,091 to 293,550. Information regarding the prevalence of methicillin resistance in staphylococcal isolates in the community was obtained from statistics on wound cultures from 3 urban emergency departments representative of the Greater Phoenix area. The percentage of SSTIs caused by MRSA was 47.8%, 46%, 43.1%, and 41.8% respectively in the 4-year study period [20].

**Figure 1:**
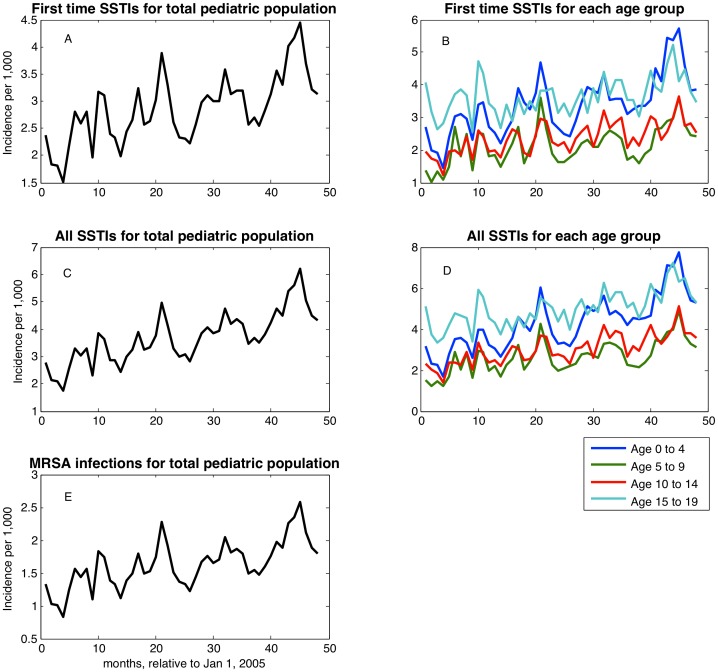
Time series of incidence of general SSTIs in total pediatric population and four pediatric age groups: first time (A and B); sum of first time and recurrent (C, D); incidence of first time and recurrent MRSA-related SSTIs in total pediatric population (E).

We stratified patients into 4 age groups: 0–4 years, 5–9 years, 10–14 years, and 15–19 years. Using the corresponding denominator population obtained from CHIR, we generated time series of monthly SSTI incidence per 1,000 people as shown in Figure 1. Of note, this incidence curve corresponds to SSTIs including MRSA. We then applied the percentage of MRSA in wound cultures for each year to derive incidence curves for MRSA infections.

### Climate data

To assess the temporal correlation between SSTI incidence and climatological variables, we obtained daily time series of mean temperature, specific humidity, and relative humidity from the National Oceanic and Atmospheric Administration records, accessible from the Weather Underground website (http://www.wunderground.com/).

### Statistical Methods

For the time-series of SSTI incidence for each age group, we used a well-known spectral analysis least-squares method known as the Lomb-Scargle periodogram [21]–[23] to determine the frequency spectrum (or period). The Lomb-Scargle method is similar to Fourier spectral analysis methods, except with the added benefit that bootstrapping methods are used to calculate the probability of false-alarm peaks in the spectrum (i.e., the bootstrapping methods determine the statistical significance of observed peaks in the spectrum). It is implemented in R language and available from http://research.stowers-institute.org/efg/2005/LombScargle. After the period 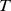 being determined, in order to estimate the overall linear trend and peak of the seasonal pattern, we fitted the following nonlinear regression model (in R language)
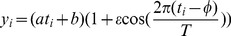
(1)where 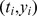 is the incidence data point, 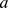 and 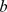 parameterize the overall linear trend, 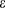 is the degree of seasonality (or the strength of the seasonal forcing), 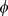 is the phase in incidence which gives the month of the year when SSTI incidence is maximal.

## Results

The monthly incidence of first time SSTIs showed an overall increasing trend with annual periodicity between January 1, 2005 and December 31, 2008 for the entire pediatric population (Figure 1A) and for each age group (Figure 1B). The overall incidence, when recurrent infections are included, shows a similar seasonality pattern and temporal trend (Figure 1C and Figure 1D). Moreover, the same pattern can be observed for monthly incidence of MRSA-related SSTIs, by applying the percentages of MRSA from wound cultures [20](Figure 1E). The overall yearly incidence of all SSTIs was 3.36%, 4.12%, 4.59% and 5.55% for the years 2005–2008 respectively.

**Figure 2:**
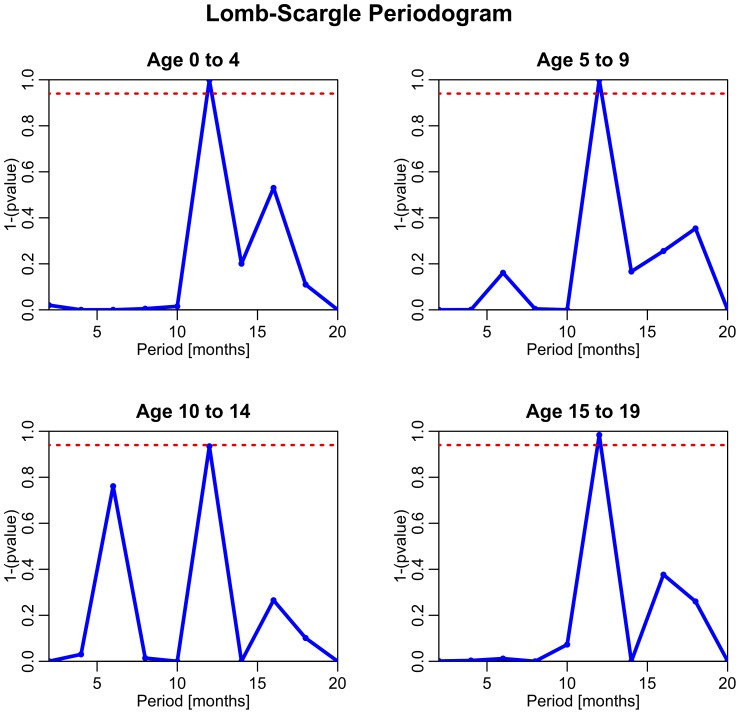
Lomb-Scargle periodograms testing the significance of cyclical patterns of periods between 2 and 20 months in the time series of incidence monthly SSTIs. Points above the red dotted line indicate the period is significant to 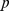
<.05.

Figure 2 shows the significance of the seasonal pattern using period lengths from 3 to 20 months across four age groups. In this analysis we consider seasonality with periods that are significant at the 95% level. We found a statistically significant ∼12 month periodic cycle in incidence for all four age groups.

**Figure 3:**
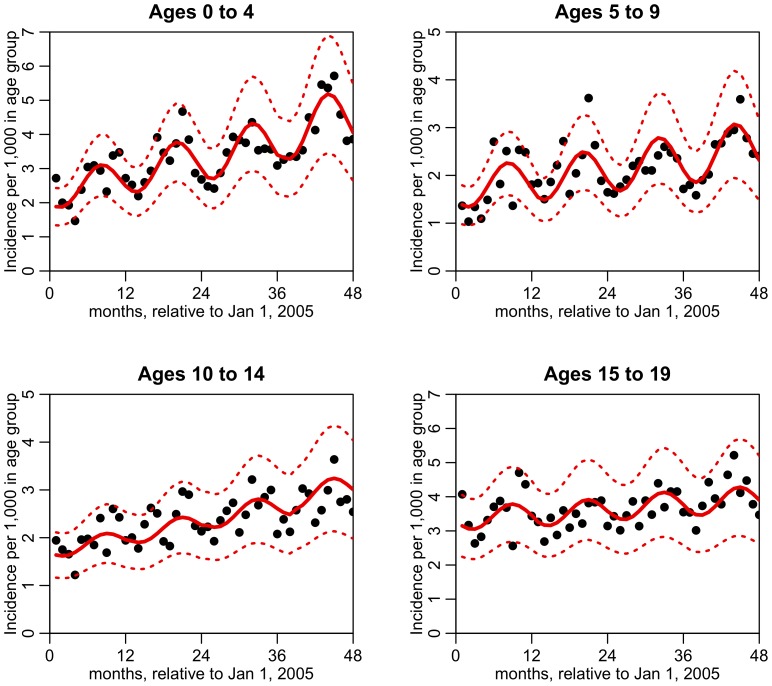
Time series of monthly SSTI incidence in four pediatric age groups (points), and the best fit of linear trend plus seasonality with period 12 months (red line). The dotted red lines indicate the 95% CI on the fit prediction.

The best-fit models for the four age groups are shown in Table 1 and Figure 3. The 95% confidence intervals for 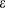 (seasonality forcing) are greater than zero for all four age groups, indicating a significant annual seasonality pattern in all four age groups. The 95% confidence intervals for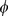 (phase of the seasonality) of the four age groups overlap, and indicate that the month of September is when SSTI incidence peaks for children of all ages. The p-value testing the hypothesis that all four values of 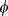 are drawn from the same mean is 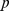 =  0.14 (Pearson two-tailed).

**Table 1:** Best-fit estimate and confidence interval for 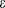
(seasonality forcing) and 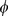
(phase of the seasonality, or where the peak of a period is).

Age group	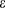	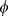
0 – 4	0.19 [0.14,0.24]	7.9 [7.4,8.4]
5 – 9	0.22 [0.16,0.29]	8.1 [7.5,8.7]
10 – 14	0.09 [0.03,0.14]	8.6 [7.4,9.8]
15 – 19	0.10 [0.04,0.15]	8.7 [7.8,9.7]

All age groups show a significant upward incidence trend. Based on the model fit of the nonlinear regression model (Equation (1)), we estimate that in 5 years (year 2013) the incidence will be 1.6 to 1.9 times greater than in 2008 for the 0 to 4 age group (95% CI). Similar but smaller increases could also be expected for other age groups, with the confidence intervals being [1.3, 1.8], [1.2,1.7], and [1.0, 1.6] in the 5 to 9, 10 to 14, and 15 to 19 age groups respectively.

**Figure 4:**
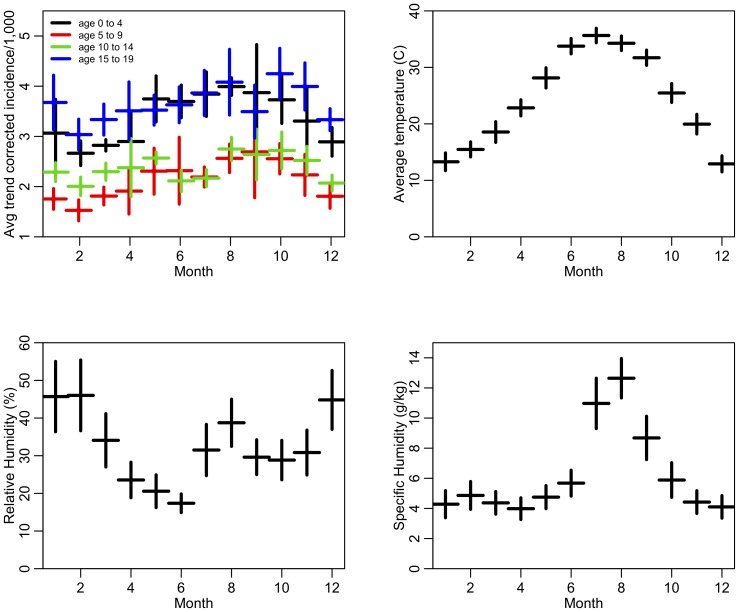
The first plot shows the within-month average of the trend-correlated incidence of SSTIs for each of the four pediatric age groups. The vertical error bars represent the one standard deviation error on the mean. The trend corrected incidence is the incidence minus the trend term in the regression model (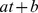 in Equation (1)). The second to fourth plots show the within-month average of temperature, relative humidity, and specific humidity, respectively.

In Figure 4 we show the within-month average of temperature, relative humidity, and specific humidity between 2005 and 2008. In all four age groups, temperature is significantly correlated to SSTI incidence (one tailed 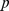
<.05). Specific humidity is also significantly correlated to incidence across all age groups except for those aged 10–14 years.

## Discussion

We have carried out a time series analysis of age-specific SSTI infections in children and adolescents < = 19 years who were enrolled in AHCCCS, the Medicaid program in Arizona. We found that the temporal trend of SSTIs follows strong annual seasonality with peak timing in incidence occurring approximately in early September. Further, all of the 4 pediatric groups in our data share this pattern. Our findings may be explained by the high correlation between skin infection and environmental factors such as temperature and specific humidity. The average temperature in Maricopa County in Arizona is as high as 38 °C in summer months (May – September), with peak average temperature in late July. Humidity in the skin from perspiration may facilitate SSTI development by increasing bacterial load on skin and making certain areas of the skin more vulnerable to bacterial infection. This may also explain regional variation in temporal peak of CA-MRSA infections as found in other studies (e.g., [14]). Another potential factor associated with this temporal pattern could be the start of school activities in early August (or early September in other parts of the country). CA-MRSA, like all strains of S *aureus*, is transmitted by direct contact with the index patient or carrier, usually by skin-to-skin contact with a colonized or infected individual. It is well documented that contact frequency among young children increases during school time compared to summer vacation period [24]. The delay of approximately one month from the start of school activities to the timing of the infection peak in early September could be explained by the intrinsic disease dynamics including the multiple stages of the MRSA infection cycle (contact-colonization-infection) and possible downward household transmission from school-aged children to their younger siblings. We also observed higher incidence in children in the age groups 0–4 year and 15–19 year. Other studies have reported similar results (e.g., [25]–[27]). The increased incidence in older adolescents could also be due to increased skin abrasions during contact sports while the increased incidence in children may be related to diaper use in this age group since a large number of MRSA abscesses start in broken skin in the diaper area.

Our study has limitations that should be noted. Our dataset captures only those cases coded as SSTI, and hence cannot directly capture the infections caused by CA-MRSA. Utilizing laboratory diagnosis of MRSA will not be accurate in a population based study including office visits, and is likely to underestimate the burden of infections caused by MRSA. Although incidence in our study is based on the total number of SSTIs, with the specific fraction caused by MRSA as determined from microbiology statistics, this represents true clinical practice at this time. That is, most cases of SSTIs were clinically diagnosed and specimens for microbiological testing were not collected. Future studies using clinical and laboratory data are warranted to corroborate our findings and pinpoint the correlation and contribution of MRSA to SSTIs.

Our study is one of the few where incidence in a population can be described, since the majority of SSTIs are treated in the offices of primary care physicians, rather than in hospitals. We chose to use only Medicaid data, since these data were the most complete in our data repository. It is important to note that patients under Medicaid coverage belong to a lower income population, where rates of infection have been described to be higher than in the general population [28], [29]. Therefore our data are likely to overestimate the actual incidence in the overall population. However, the seasonality pattern described here is likely to be representative of the general population.

Another limitation is the retrospective nature of our study. We note that the percentage of MRSA in wound cultures decreased from 2005 to 2008. This could be explained by the change in antibiotic prescription practices as health care workers became more aware of CA-MRSA, but the specific antibiotics used for treatment of each SSTI were not examined.

A clear picture of the seasonality and trend of SSTIs, especially those MRSA-related, at the population level has implications for clinical treatment and public health interventions. With this information, potential factors affecting MRSA infection and transmission could be targeted. However, the exact interaction of age, length of contact, temperature, and other factors effecting MRSA infection and transmission remain to be elucidated. Also, further studies are needed to determine the cause of the September peak in incidence. However, peak incidence timing in September could inform prevention efforts and control interventions. Of note, current treatment and decolonization (e.g., with mupirocin [30]) strategies do not change throughout the year. However, our results suggest that enhanced decolonization efforts in the patient and his/her household contacts could be conducted during the summer months as a mitigation strategy. Conversely, a less aggressive approach during the remaining part of the year could decrease the probability of emergence of further antibiotic resistance to mupirocin in CA-MRSA strains [31]. However, prospective studies examining such strategies would be needed to determine their impact on incidence and the dynamics of antibiotic resistant infections.
